# Induced magnetic field and viscous dissipation on flows of two immiscible fluids in a rectangular channel

**DOI:** 10.1038/s41598-021-03313-9

**Published:** 2022-01-07

**Authors:** Nehad Ali Shah, Hussam Alrabaiah, Dumitru Vieru, Se-Jin Yook

**Affiliations:** 1 Department of Mathematics, Lahore Leads University, Lahore, Pakistan; 2 College of Engineering, Al Ain University, Al Ain, United Arab Emirates; 3 Mathematics Department, Tafila Technical University, Tafila, Jordan; 4 Department of Theoretical Mechanics, Technical University of Iasi, Iasi, Romania; 5 School of Mechanical Engineering, Hanyang University, 222 Wangsimni-ro, Seongdong-gu, Seoul, 04763 Republic of Korea

**Keywords:** Engineering, Mathematics and computing, Physics

## Abstract

The unsteady, magneto-hydrodynamic generalized Couette flows of two immiscible fluids in a rectangular channel with isothermal walls under the influence of an inclined magnetic field and an axial electric field have been investigated. Both fluids are considered electrically conducting and the solid boundaries are electrically insulated. Approximate analytical solutions for the velocity, induced magnetic, and temperature fields have been determined using the Laplace transform method along with the numerical Stehfest's algorithm for the inversion of the Laplace transforms. Also, for the nonlinear differential equation of energy, a numerical scheme based on the finite differences has been developed. A particular case has been numerically and graphically studied to show the evolution of the fluid velocity, induced magnetic field, and viscous dissipation in both flow regions.

## Introduction

The dynamics that occur in the motion of liquid–solid, liquid–liquid and liquid–gas environments are a rich source of interdisciplinary research, for example, in nanotechnology, the nuclear industry, as well as when cooling devices in microelectronics. The overall properties and applicability of multiphase systems are totally dependent on interface shapes, which influence and are influenced by each phase's flow area, where the heat fluxes are not equal since the transition in interfacial energy is considered^1,2^.

Magneto-hydrodynamic (MHD) devices, generators, accelerators, and flow meters use the flow and heat transfer of electrically conducting fluids in channels and circular pipes under the influence of a transverse magnetic field, and have uses in nuclear reactors, filtration, and heat exchangers, etc. Decades prior, there was an involvement in the influence of the outer magnetic field on heat-physical mechanisms. Blum et al.^3^ studied heat and mass transfer in the presence of a magnetic field and that was one of the first works. Many researchers have investigated the flow and heat transfer of a viscous, incompressible, electrically conducting fluid, between two infinite, parallel, insulating plates^4–6^. Convective heat transfer in channels has also become a common research subject in recent decades due to its applications in solar energy, gas cooled reactor protection, and crystal growth in liquids, among other things. The first research into a two-phase liquid metal magneto-fluid-mechanics generator was started by Thome^7^.In this study have been analyzed effects of the magnetic field on distribution of gas in the field direction, slip ratio, and two-phase pressure drop, by using streams of sodium–potassium alloy and nitrogen that were mixed and pumped through a vertical, rectangular channel with a transverse magnetic field applied perpendicularly to the long side of the cross section. Postlethwaite and Sluyter^8^ gave a summary of the heat transfer issues that MHD generators face. Lohrasbi and Sahai^9^ investigated MHD two-phase flow and heat transfer in a horizontal parallel-plate channel and identified analytical solutions for velocity and temperature profiles where only one of the fluids is electrically conducting. Closed-form solutions for two-phase flow and heat transfer in a horizontal channel with both phases electrically conducting were mentioned by Malashetty and Leela^10,11^.

Malashetty and Umavathi^12^ recently investigated two-phase MHD flow and heat transfer in an inclined channel with buoyancy effects for the case where only one of the phases is electrically conducting. The studies mentioned above are helpful in determining the impact of slag layers on the heat transfer aspects of coal fired MHD generators.

Sai et al.^13^ investigated the flow of two immiscible fluids in a channel with one permeable wall caused by an oscillatory pressure gradient. They found slip conditions on the channel wall but assumed continuous velocities and shear stresses at the fluid–fluid interface. The authors studied the flow of water and mercury under the influence of a sinusoidal time-dependent pressure gradient as a particular case. By introducing cross terms to the generalized Darcy law, Pasquier et al.^14^ developed a statistical model of creeping flows that provides an explicit coupling term in both phases. Using an extension of the Buckley-Leverett theorem, the effect of the additional terminology on saturation profiles and pressure drops was studied. Nicodijevic et al.^15^ have studied magneto-hydrodynamic Couette flows of two immiscible, electrically conducting Newtonian fluids in a horizontal channel. The flow is influenced by an electric field and an inclined magnetic field while, the channel walls are isothermal and insulated. The induced magnetic field has been also determined. Other interesting results of immiscible fluids flow can be found in^16–18^.

Thermal management systems/devices must be able to withstand a variety of thermal boundary conditions due to their vast range of applications in various sectors. Uniform heat flux, uniform wall temperature, and insulated wall boundary conditions are common examples. Numerous studies on studying the convective heat transfer process under such boundary circumstances have been published in the literature. However, depending on the flow environment, an asymmetric thermal boundary condition may be required to investigate the system's underlying thermal transport properties. We'd like to point out that there are a few studies in the literature that analyze the thermo-hydrodynamics of both Newtonian and non-Newtonian fluids while taking the aforementioned thermal boundary conditions into account^19–21^.

The purpose of this article it to investigate the unsteady, magneto-hydrodynamic generalized Couette flows of two immiscible fluids in a rectangular channel with isothermal walls under the influence of an inclined magnetic field and an axial electric field have been investigated. Both fluids are considered electrically conducting and the solid boundaries are electrically insulated. The problem is formulated into the dimensionless form and, at the interface the velocity, shear stress, induced magnetic and temperature fields are considered continuous functions. The bottom wall of channel is fixed, while the upper wall is moving with a given time-dependent velocity. The nonslip conditions on the solid boundaries are also considered. Approximate analytical solutions for the velocity, induced magnetic, and temperature fields have been determined using the Laplace transform method along with the numerical Stehfest's algorithm for the inversion of the Laplace transforms. Also, for the nonlinear differential equation of energy, a numerical scheme based on the finite differences has been developed. A particular case characterized by a time-exponential velocity of the upper wall, has been numerically and graphically studied to show the evolution of the fluid velocity, induced magnetic field, and viscous dissipation in both flow regions.

## Problem formulation

**Figure 1:**
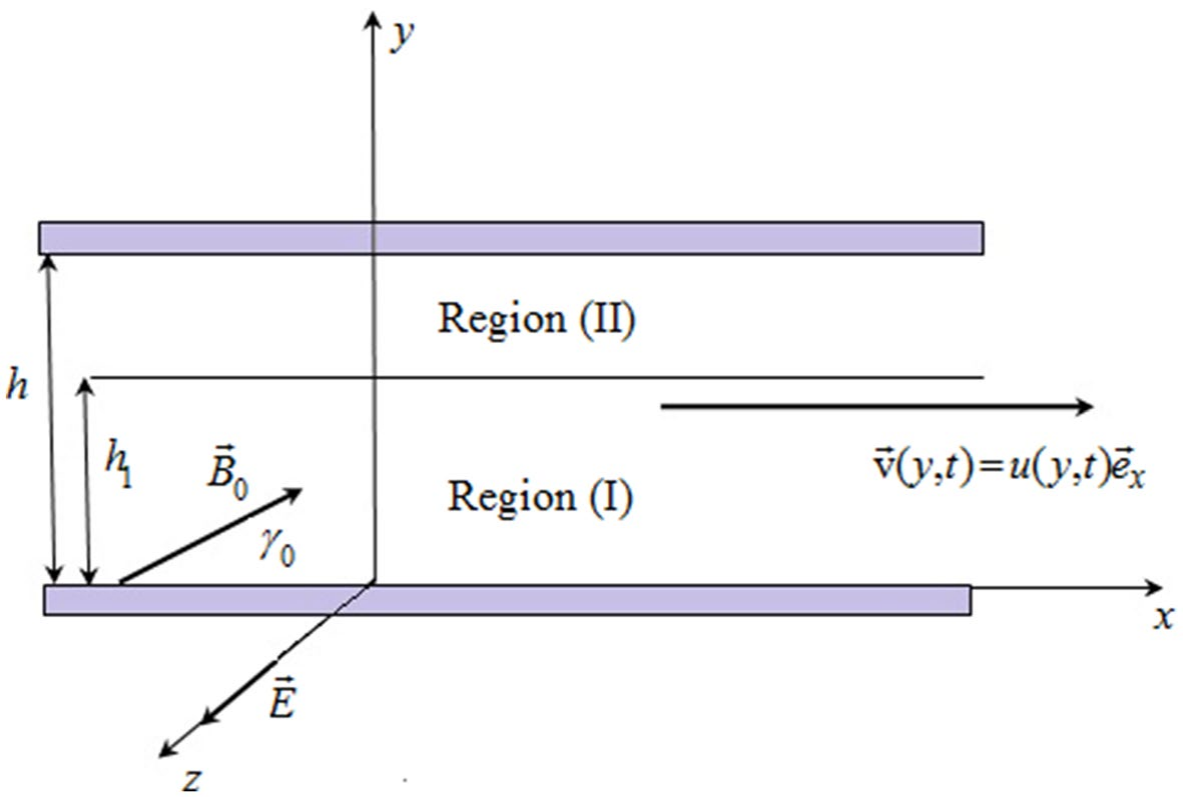
Physical model. Coordinate system.

The physical model shown in Fig. 1 consists of two infinite, parallel plates extending in the x-direction, and z-direction. The y-axis is perpendicular to the channel walls. The fluids in the two domains are assumed immiscible and electrically conducting. The flow is incompressible, unsteady, one-dimensional, and fully developed.

The region $R_{1} = \left\{ {\left( {x,y,z} \right) \in {\mathbb{R}}^{3} ,\,\,0 \le y \le h_{1} } \right\}$ is occupied by a fluid of viscosity $\mu_{1}$, density $\rho_{1}$, electrical conductivity $\sigma_{1}$, thermal conductivity $k_{1}$, specific heat capacity $c_{p1}$, and the magnetic permittivity $\mu_{e1}$.

The region $R_{2} = \left\{ {\left( {x,y,z} \right) \in {\mathbb{R}}^{3} ,\,\,h_{1} \le y \le h} \right\}$ is field by a different fluid of viscosity $\mu_{2}$, density $\rho_{2}$, electrical conductivity $\sigma_{2}$, thermal conductivity $k_{2}$, specific heat capacity $c_{p2}$, and the magnetic permittivity $\mu_{e2}$.

It is supposed that fluids have laminar, one-dimensional flow with the velocity fields.

1 $$\vec{V}_{i} \left( {y,t} \right) = u_{i} \left( {y,t} \right)\vec{e}_{x} {,}\quad \, i = 1,2,$$

where $\vec{e}_{x}$ is the unit vector along the x-direction.

A uniform magnetic field of the strength ${\rm B}_{0}$ is applied such that the angle between vectors $\vec{e}_{x} {\text{ and }}\vec{\rm B}_{0}$ is equal to $\gamma_{0} \in (0,\,\left. {\pi /2} \right]\,,$ therefore, the applied magnetics vector field is

2 $$\vec{\rm B}_{0} = {\rm B}_{0} \cos \gamma_{0} \vec{e}_{x} { + }{\rm B}_{0} \sin \gamma_{0} \vec{e}_{y} .$$

Due to the fluid motion, a magnetic field is induced along of the lines of motion, namely

3 $$\vec{\rm B}_{1} = b(y,t)\vec{e}_{x} ,$$

is induced along of the lines of motion.

The total magnetic field is given by

4 $$\vec{\rm B} = \vec{\rm B}_{0} + \vec{\rm B}_{1} = \left( {b(y,t) + {\rm B}_{0} \cos \gamma_{0} } \right)\vec{e}_{x} { + }{\rm B}_{0} \sin \gamma_{0} \vec{e}_{y} .$$

The walls of the rectangular channel are kept at the constant temperature $T_{{w_{0} }} \,{\text{and }}T_{{w_{1} }} ,$ respectively and the plates are electrically insulated.

Also, a time-dependent electric field

5 $$\vec{E} = E_{0} \sin (\omega t)\vec{e}_{z} ,$$

is applied to the fluids.

In the above hypotheses, the continuity equation $\nabla \cdot \vec{V}_{i} = 0$ is satisfied by the velocity fields in (1).

The electro-magneto-hydrodynamic fluids flow problem is mathematically described by^22–24^:

The momentum equation

6 $$\rho \left( {\frac{{\partial \vec{V}}}{\partial t} + \left( {\vec{V} \cdot \nabla } \right)\vec{V}} \right) = - \nabla p + \vec{J} \times \vec{B},$$

The constitutive equation

7 $$S = \mu \left( {\nabla \vec{V} + \left( {\nabla \vec{V}} \right)^{T} } \right),$$

The magnetic induction equation

8 $$\frac{{\partial \vec{B}}}{\partial t} - \nabla \times \left( {\vec{V} \times \vec{B}} \right) - \frac{1}{{\sigma \mu_{e} }}\Delta \vec{B} = 0,$$

The energy equation

9 $$\rho c_{p} \left( {\frac{\partial T}{{\partial t}} + \vec{V}\nabla T} \right) = k\Delta T + \mu \phi + \frac{1}{\sigma }\vec{J}^{2} ,$$

where, the dissipation function $\phi$ is given by

10 $$\phi = 2\left[ {\left( {\frac{\partial u}{{\partial x}}} \right)^{2} + \left( {\frac{\partial v}{{\partial y}}} \right)^{2} + \left( {\frac{\partial w}{{\partial z}}} \right)^{2} } \right] + \left( {\frac{\partial v}{{\partial x}} + \frac{\partial u}{{\partial y}}} \right)^{2} + \left( {\frac{\partial w}{{\partial y}} + \frac{\partial v}{{\partial z}}} \right)^{2} + \left( {\frac{\partial u}{{\partial z}} + \frac{\partial w}{{\partial x}}} \right)^{2} - \frac{2}{3}\left( {\nabla \vec{V}} \right)^{2} = \left( {\frac{\partial u}{{\partial y}}} \right)^{2} ,$$

and the current density vector due to the magnetic field and electric field is

11 $$\vec{J} = \sigma \left( {\vec{E} + \vec{V} \times \vec{B}} \right) = \sigma \left( {E_{0} sin(\omega t) + {\rm B}_{0} \sin \gamma_{0} u(y,t)} \right)\vec{e}_{z} .$$

The magnetic body force becomes

12 $$\begin{aligned} \vec{F}_{m} & = \vec{J} \times \vec{B} = - \sigma {\rm B}_{0} \sin \gamma_{0} \left( {E_{0} sin(\omega t) + {\rm B}_{0} \sin (\gamma_{0} )u(y,t)} \right)\vec{e}_{x} \\ & \quad + \sigma \left( {b(y,t) + {\rm B}_{0} \cos \gamma_{0} } \right)\left( {E_{0} sin(\omega t) + {\rm B}_{0} \sin (\gamma_{0} )u(y,t)} \right)\vec{e}_{y} . \\ \end{aligned}$$

In the case of $\vec{V} = u(y,t)\vec{e}_{x}$, the above equations reduce to

13 $$\rho \frac{\partial u(y,t)}{{\partial t}} = - \frac{\partial p}{{\partial x}} + \frac{{\partial S_{xy} (y,t)}}{\partial y} - \sigma {\rm B}_{0} \sin \gamma_{0} \left( {E_{0} sin(\omega t) + {\rm B}_{0} \sin (\gamma_{0} )u(y,t)} \right),$$

14 $$0 = - \frac{\partial p}{{\partial y}} + \sigma \left( {b(y,t) + {\rm B}_{0} \cos \gamma_{0} } \right)\left( {E_{0} sin(\omega t) + {\rm B}_{0} \sin (\gamma_{0} )u(y,t)} \right),$$

15 $$S_{xy} (y,t) = \mu \frac{\partial u(y,t)}{{\partial t}},$$

16 $$\frac{\partial b(y,t)}{{\partial t}} - {\rm B}_{0} \sin \gamma_{0} \frac{\partial u(y,t)}{{\partial y}} - \frac{1}{{\sigma \mu_{e} }}\frac{{\partial^{2} b(y,t)}}{{\partial y^{2} }} = 0,$$

17 $$\rho c_{p} \left( {\frac{\partial T}{{\partial t}} + u\frac{\partial T}{{\partial x}}} \right) = k\Delta T + \mu \left( {\frac{\partial u}{{\partial y}}} \right)^{2} + \sigma \left( {E_{0} sin(\omega t) + {\rm B}_{0} \sin \gamma_{0} u} \right)^{2} .$$

In this paper, we shall study the problem for zero pressure gradient in the x-direction namely $\frac{\partial p}{{\partial x}} = 0$. Also, the Eq. (14) that gives the pressure gradient in the y-direction will not be discussed, because it is clear that if $b(y,t){\text{ and u}}(y,t)$ are known the variation of the pressure *p* is easily determined.

The flow, induced magnetic and temperature field of the fluid in two regions are determined by the equations

18 $$\rho_{i} \frac{{\partial u_{i} (y,t)}}{\partial t} = \frac{{\partial S_{xy}^{i} (y,t)}}{\partial y} - \sigma_{i} {\rm B}_{0} \sin \gamma_{0} \left( {E_{0} sin(\omega t) + {\rm B}_{0} \sin (\gamma_{0} )u_{i} (y,t)} \right),$$

19 $$S_{xy}^{i} (y,t) = \mu_{i} \frac{{\partial u_{i} (y,t)}}{\partial y},$$

20 $$\frac{{\partial b_{i} (y,t)}}{\partial t} - \frac{1}{{\sigma_{i} \mu_{ei} }}\frac{{\partial^{2} b_{i} (y,t)}}{{\partial y^{2} }} = {\rm B}_{0} \sin \gamma_{0} \frac{{\partial u_{i} (y,t)}}{\partial y},$$

21 $$\rho_{i} c_{pi} \left( {\frac{{\partial T_{i} }}{\partial t} + u_{i} \frac{{\partial T_{i} }}{\partial x}} \right) = k_{i} \Delta T_{i} + \mu_{i} \left( {\frac{{\partial u_{i} }}{\partial y}} \right)^{2} + \sigma_{i} \left( {E_{0} sin(\omega t) + {\rm B}_{0} \sin \gamma_{0} u_{i} } \right)^{2} ;\,\quad \,\,i = 1,2.$$

Along with Eqs. (18)–(21), the following initial, boundary and interface conditions are considered:

22 $$u_{1} (y,0) = 0, \, u_{2} (y,0) = 0, \, b_{1} (y,0) = b_{2} \left( {y,0} \right) = 0, \, T_{1} (x,y,0) = T_{{w_{o} }} = T_{2} (x,y,0),$$

23 $$u_{1} (0,t) = 0, \, u_{2} (h,t) = U_{0} f_{2} (t),\,\,U_{0} { > }\,{0, }\left( {\text{No slip conditions}} \right),$$

24 $$u_{1} (h_{1} ,t) = \, u_{2} (h_{1} ,t),$$

25 $$S^{1}_{xy} \left( {h_{1} ,t} \right) = \, S^{2}_{xy} \left( {h_{1} ,t} \right),$$

26 $$b_{1} \left( {0,t} \right) = 0,{\text{ b}}_{2} \left( {h,t} \right) = 0,$$

27 $$b_{1} \left( {h_{1} ,t} \right) = {\text{ b}}_{2} \left( {h_{1} ,t} \right),$$

28 $$\frac{1}{{\sigma_{1} \mu_{e1} }}\left. {\frac{{\partial b_{1} (y,t)}}{\partial y}} \right|_{{y = h_{1} }} = \frac{1}{{\sigma_{2} \mu_{e2} }}\left. {\frac{{\partial b_{2} (y,t)}}{\partial y}} \right|_{{y = h_{1} }} ,$$

29 $$T_{1} \left( {x,0,t} \right) = T_{{w_{0} }} {\text{, T}}_{2} \left( {x,h,t} \right) = T_{{w_{1} }}$$

30 $$T_{1} \left( {x,h_{1} ,t} \right) = {\text{T}}_{2} \left( {x,h_{1} ,t} \right),$$

31 $$k_{1} \left. {\frac{{\partial T_{1} }}{\partial y}} \right|_{{y = h_{1} }} = k_{2} \left. {\frac{{\partial T_{2} }}{\partial y}} \right|_{{y = h_{1} }} .$$

In the present problem, it has assumed that the two plates are maintained at constant temperatures. Under the flow's assumptions, the temperature fields can be considered independent of the longitudinal variable x. The term involving $\frac{\partial T}{{\partial x}} = 0$ in the energy Eq. (21) drops out for such conditions.

We introduce the non-dimensional entities

32 $$\begin{gathered} \tilde{u}_{i} = \frac{{u_{i} }}{{U_{0} }},{\tilde{\text{y}}} = \frac{y}{{h_{1} }},{\tilde{\text{x}}} = \frac{x}{{h_{1} }},{\text{ d}} = \frac{h}{{h_{1} }},{\tilde{\text{t}}} = \frac{{U_{0} t}}{{h_{1} }}, \, \tilde{S}^{i}_{{\tilde{x}\tilde{y}}} = \frac{{S_{xy}^{i} }}{{\rho_{i} U^{2}_{0} }}, \, \tilde{\omega } = \frac{{h_{1} \omega }}{{U_{0} }},\,\,E_{i} = \frac{{\sigma_{i} B_{0} E_{0} h_{1} \sin \gamma_{0} }}{{\rho_{i} U_{0}^{2} }}, \hfill \\ F_{i} = \frac{{\sigma_{i} B_{0}^{2} h_{1} \sin^{2} \gamma_{0} }}{{\rho_{i} U_{i} }},{\text{ Re}}_{i} = \frac{{h_{1} U_{0} }}{{\nu_{i} }},\,\,\tilde{b}_{i} = \frac{{b_{i} }}{{B_{0} \sin \gamma_{0} }}, \, \theta_{i} = \frac{{T_{i} - T_{{w_{0} }} }}{{T_{{w_{1} }} - T_{{w_{0} }} }},{\text{ Pr}}_{i} = \frac{{\mu_{i} c_{pi} }}{{k_{i} }},{\text{ Ec}}_{i} = \frac{{U_{0}^{2} }}{{c_{pi} (T_{{w_{1} }} - T_{{w_{0} }} )}}, \hfill \\ Ha_{i} = B_{0} h_{1} \sqrt {\frac{{\sigma_{i} }}{{\mu_{i} }}} ,{\text{ G}}_{0} = \frac{{E_{0} }}{{B_{0} U_{0} \sin \gamma_{0} }}, \, \tilde{f}\left( {\tilde{t}} \right) = f_{2} \left( {\frac{{h_{1} }}{{U_{0} }}\tilde{t}} \right),\delta_{0} = \frac{{\mu_{e1} \sigma_{1} }}{{\mu_{e2} \sigma_{2} }},k_{0} = \frac{{k_{2} }}{{k_{1} }},Rm_{i} = U_{0} h_{1} \sigma_{i} \mu_{ei} . \hfill \\ \end{gathered}$$

Equations (18)–(21) become (the notation “$\sim$” is neglected)

33 $$\frac{{\partial u_{i} (y,t)}}{\partial t} = \frac{{\partial S_{xy}^{i} (y,t)}}{\partial y} - E_{i} sin(\omega t) - F_{i} u_{i} (y,t),$$

34 $$S_{xy}^{i} (y,t) = \frac{1}{{{\text{Re}}_{i} }}\frac{{\partial u_{i} (y,t)}}{\partial y},$$

35 $$\frac{{\partial b_{i} (y,t)}}{\partial t} - \frac{1}{{Rm_{i} }}\frac{{\partial^{2} b_{i} (y,t)}}{{\partial y^{2} }} = \frac{{\partial u_{i} (y,t)}}{\partial y}$$

36 $$\Pr_{i} {\text{Re}}_{i} \frac{{\partial \theta_{i} }}{\partial t} = \frac{{\partial^{2} \theta {}_{i}}}{{\partial y^{2} }} + \Pr_{i} Ec_{i} \left( {\frac{{\partial u_{i} }}{\partial y}} \right)^{2} + \Pr_{i} Ec_{i} Ha_{i}^{2} \sin^{2} \gamma_{0} \left( {G_{0} sin(\omega t) + u_{i} } \right)^{2} .$$

The non-dimensional boundary conditions are

37 $$u_{i} \left( {y,0} \right) = 0, \, b_{i} \left( {y,0} \right) = 0, \, \theta_{i} \left( {y,0} \right) = 0, \, i = 1,2, \, y \in \left[ {0,d} \right],$$

38 $$\begin{gathered} u_{1} \left( {0,t} \right) = 0, \, u_{2} \left( {d,t} \right) = f\left( t \right), \hfill \\ u_{1} \left( {1,t} \right) = u_{2} \left( {1,t} \right), \hfill \\ S_{xy}^{1} \left( {1,t} \right) = \, S_{xy}^{2} \left( {1,t} \right), \hfill \\ \end{gathered}$$

39 $$\begin{gathered} b_{1} \left( {0,t} \right) = 0, \, b_{2} \left( {d,t} \right) = 0, \hfill \\ b_{1} \left( {1,t} \right) = b_{2} \left( {1,t} \right), \hfill \\ \left. {\frac{{\partial b_{1} (y,t)}}{\partial y}} \right|_{y = 1} = \left. {\delta_{0} \frac{{\partial b_{2} (y,t)}}{\partial y}} \right|_{y = 1} , \hfill \\ \end{gathered}$$

40 $$\begin{gathered} \theta_{1} \left( {0,t} \right) = 0, \, \theta_{2} \left( {d,t} \right) = 1, \hfill \\ \theta_{1} \left( {1,t} \right) = \theta_{2} \left( {1,t} \right), \hfill \\ \left. {\frac{{\partial \theta_{1} \left( {y,t} \right)}}{\partial y}} \right|_{y = 1} = k_{0} \left. {\frac{{\partial \theta_{2} \left( {y,t} \right)}}{\partial y}} \right|_{y = 1} . \hfill \\ \end{gathered}$$

## Velocity field distribution

To solve Eqs. (33) and (34) along with initial-boundary conditions (37) and (38), we use the Laplace transform method. Applying the Laplace transform to Eqs. (33) and (34), we obtain

41 $$s{\overline{u}}_{i} (y,s) = \frac{{\partial {\overline{S}}^{i}_{xy} (y,t)}}{\partial y} - E_{i} \frac{\omega }{{s^{2} + \omega^{2} }} - F_{i} {\overline{u}}_{i} \left( {y,s} \right),$$

42 $${\overline{S}}^{i}_{xy} (y,s) = \frac{1}{{{\text{Re}}_{i} }}\frac{{\partial {\overline{u}}_{i} (y,s)}}{\partial y},$$

where, $\overline{\chi }(y,s) = \int_{0}^{\infty } {\chi (y,t)\exp ( - st)} ds$ denotes the Laplace transform of function $\chi (y,t)$.

Eliminating ${\overline{S}}_{xy}^{i}$ between Eqs. (41) and (42), we obtain the following equation for the Laplace transform ${\overline{u}}_{i} \left( {y,s} \right)$ of the velocity $u_{i} (y,t),\;i = 1,2$:

43 $$\frac{{\partial^{2} {\overline{u}}_{i} (y,s)}}{{\partial y^{2} }} = {\text{Re}}_{i} \left( {s + F_{i} } \right){\overline{u}}_{i} \left( {y,s} \right) + \frac{{E_{i} {\text{Re}}_{i} \omega }}{{s^{2} + \omega^{2} }}, \, i = 1,2.$$

Functions ${\overline{u}}_{i} \left( {y,s} \right)$ have to satisfy the boundary and interface conditions

44 $$\begin{gathered} {\overline{u}}_{1} \left( {0,s} \right) = 0, \, {\overline{u}}_{2} \left( {d,s} \right) = {\overline{f}}\left( s \right), \hfill \\ {\overline{u}}_{1} \left( {1,s} \right) = {\overline{u}}_{2} \left( {1,s} \right), \hfill \\ \left. {\frac{1}{{{\text{Re}}_{1} }}\frac{{\partial {\overline{u}}_{1} (y,s)}}{\partial y}} \right|_{y = 1} = \left. {\frac{1}{{{\text{Re}}_{2} }}\frac{{\partial {\overline{u}}_{2} (y,s)}}{\partial y}} \right|_{y = 1} . \hfill \\ \end{gathered}$$

The general solution of Eq. (43) is

45 $${\overline{u}}_{i} \left( {y,s} \right) = C_{i1} (s)\sinh \left( {{\overline{a}}_{i} (s)y} \right) + {\overline{C}}_{i2} (s)cosh\left( {{\overline{a}}_{i} (s)y} \right) + b_{i} (s),$$

where

46 $${\overline{a}}_{i} (s) = \sqrt {{\text{Re}}_{i} \left( {s + F_{i} } \right)} , \, b_{0i} (s) = \frac{{E_{i} \omega }}{{\left( {s + F_{i} } \right)(s^{2} + \omega^{2} )}}, \, i = 1,\,2.$$

Using (44), the following expressions of the functions $C_{i1} (s),\;C_{i2} (s),\;i = 1,2$ are found:

47 $$\begin{aligned} {\overline{C}}_{11} (s) & = \frac{{\left( {{\overline{f}}(s) - {\overline{b}}_{02} (s)} \right)\cosh \left( {{\overline{a}}_{2} (s)} \right)}}{{\cosh \left( {{\overline{a}}_{2} (s)d} \right)\sinh \left( {{\overline{a}}_{1} (s)} \right)}} + \frac{{{\overline{b}}_{02} (s) - {\overline{b}}_{01} (s) + {\overline{b}}_{01} (s)cosh\left( {{\overline{a}}_{1} (s)} \right)}}{{sinh\left( {{\overline{a}}_{1} (s)} \right)}} \\ & \quad - \frac{{\sinh ((d - 1){\overline{a}}_{2} (s))}}{{\cosh \left( {{\overline{a}}_{2} (s)d} \right)\sinh \left( {{\overline{a}}_{1} (s)} \right)}}{\overline{C}}_{21} (s), \\ {\overline{C}}_{12} (s) & = - b_{01} (s), \\ {\overline{C}}_{22} (s) & = \frac{{{\overline{f}}(s) - {\overline{b}}_{02} (s)}}{{\cosh \left( {{\overline{a}}_{2} (s)d} \right)}} - \frac{{\sinh \left( {{\overline{a}}_{2} (s)d} \right)}}{{\cosh \left( {{\overline{a}}_{2} (s)d} \right)}}{\overline{C}}_{21} (s), \\ {\overline{C}}_{21} & = \frac{Fa(s)}{{Fb(s)}}, \\ Fa(s) & = {\text{Re}}_{2} {\overline{a}}_{1} (s)\left[ {{\overline{b}}_{01} (s)\cosh \left( {{\overline{a}}_{2} (s)d} \right) + \left( {{\overline{f}}(s) - {\overline{b}}_{02} (s)} \right)\cosh \left( {{\overline{a}}_{1} (s)} \right)\cosh \left( {{\overline{a}}_{2} (s)} \right)} { + \left( {{\overline{b}}_{02} (s) - {\overline{b}}_{01} (s)} \right)\cosh \left( {{\overline{a}}_{1} (s)} \right)\cosh \left( {{\overline{a}}_{2} (s)d} \right)} \right] - {\text{Re}}_{1} {\overline{a}}_{2} (s)\left( {{\overline{f}}(s) - {\overline{b}}_{02} (s)} \right)\sinh \left( {{\overline{a}}_{1} (s)} \right)\sinh \left( {{\overline{a}}_{2} (s)} \right), \\ Fb(s) & = {\text{Re}}_{1} {\overline{a}}_{2} (s)\cosh \left( {{\overline{a}}_{1} (s)} \right)sinh\left[ {\left( {d - 1} \right){\overline{a}}_{2} (s)} \right] + {\text{Re}}_{2} {\overline{a}}_{1} (s)\sinh \left( {{\overline{a}}_{1} } \right)\cosh \left[ {\left( {d - 1} \right){\overline{a}}_{2} (s)} \right]. \\ \end{aligned}$$

Although the inverse Laplace transform of the function (45) can be obtained using the residue theorem, the analytical expression of the $u(y,t)$ is complicated and difficult to be used for numerical evaluation. For this reason, we prefer to obtain the numerical values of the inverse Laplace transform of ${\overline{u}}(y,s)$ using Stehfest’s numerical algorithm^25,26^

According to the Stehfest’s algorithm, the approximate values of function $u_{i} (y,t)$ are given by

48 $$u_{i} (y,t) \simeq \frac{\ln 2}{t}\sum\limits_{k = 1}^{M} {b_{k} } {\overline{u}}_{i} \left( {y,\frac{k\ln 2}{t}} \right){,}$$

where

49 $$b_{k} = ( - 1)^{{k + \frac{M}{2}}} \sum\limits_{{j = \left[ {\frac{k + 1}{2}} \right]}}^{{\min \left( {k,\frac{M}{2}} \right)}} {\frac{{j^{{{\raise0.5ex\hbox{$\scriptstyle M$} \kern-0.1em/\kern-0.15em \lower0.25ex\hbox{$\scriptstyle 2$}}}} (2j)}}{{\left( {{\raise0.5ex\hbox{$\scriptstyle M$} \kern-0.1em/\kern-0.15em \lower0.25ex\hbox{$\scriptstyle 2$}} - j} \right)!j!\left( {j - 1} \right)!\left( {k - j} \right)!\left( {2j - k} \right)!}}{.}}$$

In the above relation $M$ is an even, positive integer number and $\left[ x \right]$ denotes the integer part of the real number $x$.

## The induced magnetic field

The magnetic field induction is given by Eq. (35) along with conditions (39). Applying the Laplace transform to Eq. (35), using initial conditions (37)_2_ and the velocity expression (45), we obtain for the Laplace transform ${\overline{b}}_{i} \left( {y,s} \right)$ of $b_{i} \left( {y,t} \right)$ the differential equation

50 $$\frac{{\partial^{2} {\overline{b}}_{i} \left( {y,s} \right)}}{{\partial y^{2} }} - Rm_{i} s{\overline{b}}_{i} \left( {y,s} \right) = - Rm_{i} {\overline{a}}_{i} (s)\left[ {{\overline{C}}_{i1} (s)\cosh \left( {{\overline{a}}_{i} (s)y} \right) + {\overline{C}}_{i2} (s)sinh\left( {{\overline{a}}_{i} (s)y} \right)} \right],$$

along with the boundary and interface conditions

51 $$\begin{gathered} {\overline{b}}_{1} \left( {0,s} \right) = 0, \, {\overline{b}}_{2} \left( {d,s} \right) = 0, \, \hfill \\ {\overline{b}}_{1} \left( {1,s} \right) = {\overline{b}}_{2} \left( {1,s} \right), \hfill \\ \frac{{\partial {\overline{b}}_{1} \left( {y,s} \right)}}{\partial y}\left| {_{y = 1} } \right. = \delta_{0} \frac{{\partial {\overline{b}}_{2} \left( {y,s} \right)}}{\partial y}\left| {_{y = 1} } \right.. \hfill \\ \end{gathered}$$

The general solution of Eq. (50) is given by

52 $$\begin{aligned} {\overline{b}}_{i} \left( {y,s} \right) & = {\overline{D}}_{i1} (s)\sinh \left( {y\sqrt {Rm_{i} s} } \right) + {\overline{D}}_{i2} (s)\cosh \left( {y\sqrt {Rm_{i} s} } \right) \\ & \quad + {\overline{d}}_{i} (s)\left[ {{\overline{C}}_{i1} (s)\cosh \left( {{\overline{a}}_{i} (s)y} \right) + {\overline{C}}_{i2} (s)sinh\left( {{\overline{a}}_{i} (s)y} \right)} \right], \\ \end{aligned}$$

where

53 $${\overline{d}}_{i} (s) = \frac{{Rm_{i} {\overline{a}}_{i} (s)}}{{Rm_{i} s - {\overline{a}}_{i}^{2} (s)}} = \frac{{Rm_{i} \sqrt {{\text{Re}}_{i} \left( {s + F_{i} } \right)} }}{{\left( {Rm_{i} - {\text{Re}}_{i} } \right)s - {\text{Re}}_{i} F_{i} }}.$$

We introduce the notations

54 $${\overline{G}}_{i} \left( {y,s} \right) = {\overline{d}}_{i} (s)\left[ {{\overline{C}}_{i1} (s)\cosh \left( {{\overline{a}}_{i} (s)y} \right) + {\overline{C}}_{i2} (s)sinh\left( {{\overline{a}}_{i} (s)y} \right)} \right],$$

55 $${\overline{H}}_{i} \left( {y,s} \right) = \frac{{\partial {\overline{G}}_{i} \left( {y,s} \right)}}{\partial y} = {\overline{a}}_{i} (s){\overline{d}}_{i} (s)\left[ {{\overline{C}}_{i1} (s)sinh\left( {{\overline{a}}_{i} (s)y} \right) + {\overline{C}}_{i2} (s)cosh\left( {{\overline{a}}_{i} (s)y} \right)} \right],$$

we have

56 $$\begin{aligned} {\overline{b}}_{1} (y,s) & = {\overline{D}}_{i1} (s)\sinh \left( {y\sqrt {Rm_{i} s} } \right) + {\overline{D}}_{i2} (s)\sinh \left( {y\sqrt {Rm_{i} s} } \right) + {\overline{G}}_{i} (y,s), \\ \frac{{\partial {\overline{b}}_{1} (y,s)}}{\partial y} & = \sqrt {Rm_{i} s} \left[ {{\overline{D}}_{i1} (s)cosh\left( {y\sqrt {Rm_{i} s} } \right) + {\overline{D}}_{i2} (s)\sinh \left( {y\sqrt {Rm_{i} s} } \right)} \right] + {\overline{H}}_{i} (y,s). \\ \end{aligned}$$

Using the boundary and interface conditions (51), we obtain the algebraic system for the unknown functions ${\overline{D}}_{i1} \left( s \right),\,\,\,{\overline{D}}_{i1} \left( s \right),\,\,i = 1,\,2:$

57 $$\begin{gathered} {\overline{D}}_{12} (s) + {\overline{G}}_{1} (0,s) = 0, \hfill \\ {\overline{D}}_{21} (s)\sinh \left( {d\sqrt {Rm_{2} s} } \right) + {\overline{D}}_{22} (s)\cosh \left( {d\sqrt {Rm_{2} s} } \right) + {\overline{G}}_{2} \left( {d,s} \right) = 0, \hfill \\ {\overline{D}}_{11} (s)\sinh \left( {\sqrt {Rm_{1} s} } \right) + {\overline{D}}_{12} (s)\cosh \left( {\sqrt {Rm_{1} s} } \right) + {\overline{G}}_{1} \left( {1,s} \right) \hfill \\ \quad = \,{\overline{D}}_{21} (s)\sinh \left( {\sqrt {Rm_{2} s} } \right) + {\overline{D}}_{22} (s)\cosh \left( {\sqrt {Rm_{2} s} } \right) + {\overline{G}}_{2} \left( {1,s} \right), \hfill \\ \sqrt {Rm_{1} s} \left[ {{\overline{D}}_{11} (s)cosh\left( {\sqrt {Rm_{1} s} } \right) + {\overline{D}}_{12} (s)sinh\left( {\sqrt {Rm_{1} s} } \right)} \right] + {\overline{H}}_{1} \left( {1,s} \right) \hfill \\ \quad = \,\delta_{0} \sqrt {Rm_{2} s} \left[ {{\overline{D}}_{21} (s)cosh\left( {\sqrt {Rm_{2} s} } \right) + {\overline{D}}_{22} (s)sinh\left( {\sqrt {Rm_{2} s} } \right)} \right] + \delta_{0} {\overline{H}}_{2} \left( {1,s} \right). \hfill \\ \end{gathered}$$

whose solution is

58 $${\overline{D}}_{12} (s) = - {\overline{G}}_{1} (0,s) = - {\overline{d}}_{1} (s){\overline{C}}_{11} (s),$$

59 $${\overline{D}}_{22} (s) = \frac{{ - {\overline{G}}_{2} (d,s)}}{{\cosh \left( {d\sqrt {Rm_{2} s} } \right)}} - \frac{{\sinh \left( {d\sqrt {Rm_{2} s} } \right)}}{{\cosh \left( {d\sqrt {Rm_{2} s} } \right)}}\,{\overline{D}}_{21} (s),$$

60 $${\overline{D}}_{11} (s) = {\overline{D}}_{00} (s) + {\overline{D}}_{01} (s){\overline{D}}_{21} (s),$$

where

61 $$\begin{aligned} {\overline{D}}_{00} (s) & = {\overline{G}}_{1} (1,s)\frac{{\cosh \left( {\sqrt {Rm_{1} s} } \right)}}{{sinh\left( {\sqrt {Rm_{1} s} } \right)}} + \frac{{{\overline{G}}_{2} (1,s) - {\overline{G}}_{1} (1,s)}}{{sinh\left( {\sqrt {Rm_{1} s} } \right)}} - \frac{{{\overline{G}}_{2} (d,s)\cosh \left( {\sqrt {Rm_{2} s} } \right)}}{{sinh\left( {\sqrt {Rm_{1} s} } \right)\cosh \left( {d\sqrt {Rm_{2} s} } \right)}}, \\ {\overline{D}}_{01} (s) & = \frac{{\sinh \left( {\sqrt {Rm_{2} s} } \right)\cosh \left( {d\sqrt {Rm_{2} s} } \right) - \sinh \left( {d\sqrt {Rm_{2} s} } \right)}}{{\sinh \left( {\sqrt {Rm_{1} s} } \right)\cosh \left( {d\sqrt {Rm_{2} s} } \right)}}, \\ \end{aligned}$$

62 $${\overline{D}}_{21} (s) = \frac{{{\overline{E}}_{01} (s)}}{{{\overline{E}}_{00} (s)}},$$

with

63 $$\begin{aligned} {\overline{E}}_{00} \left( s \right) & = {\overline{D}}_{01} \sqrt {Rm_{1} s} \cosh \left( {\sqrt {Rm_{1} s} } \right) - \delta_{0} \sqrt {Rm_{2} s} \frac{{\cosh \left[ {\left( {d - 1} \right)\sqrt {Rm_{2} s} } \right]}}{{\cosh \left( {d\sqrt {Rm_{2} s} } \right)}}, \\ {\overline{E}}_{01} (s) & = \sqrt {Rm_{1} s} sinh\left( {\sqrt {Rm_{1} s} } \right){\overline{G}}_{1} \left( {0,s} \right) - {\overline{H}}_{1} \left( {1,s} \right) + \delta_{0} {\overline{H}}_{2} \left( {1,s} \right) \\ & \quad - \sqrt {Rm_{1} s} \cosh \left( {\sqrt {Rm_{1} s} } \right){\overline{D}}_{00} (s) - \frac{{\delta_{0} \sqrt {R_{m2} s} \sinh \left( {\sqrt {R_{m2} s} } \right){\overline{G}}_{2} \left( {d,s} \right)}}{{\cosh \left( {d\sqrt {R_{m2} s} } \right)}}. \\ \end{aligned}$$

Numerical values of the induced magnetic fields (52) shall be determined with the numerical approximate formula (48) updated for $b_{i} (y,t)$.

## The temperature field

### Approximate analytical solution

Let’s introduce notation

64 $$W_{i} (y,t) = \Pr_{i} Ec_{i} \left( {\frac{{\partial u_{i} (y,t)}}{\partial y}} \right)^{2} + \Pr_{i} Ec_{i} Ha_{i}^{2} \sin^{2} \gamma_{0} \left[ {G_{0} \sin (\omega t) + u_{i} (y,t)} \right]^{2} ,\,\quad \,i = 1,\,2,$$

where $u_{i}$ is given by Eq. (45).

Temperature fields are solutions of the equations

65 $$\frac{{\partial^{2} \theta_{i} (y,t)}}{{\partial y^{2} }} - \Pr_{i} {\text{Re}}_{i} \frac{{\partial \theta_{i} (y,t)}}{\partial t} + W_{i} (y,t) = 0,\,\quad i = 1,2,$$

along with the initial-boundary and interface conditions

66 $$\theta_{i} \left( {y,0} \right) = 0, \, y \in [0,d],$$

67 $$\theta_{1} \left( {0,t} \right) = 0,\,\,\,\theta_{2} \left( {d,t} \right) = 1,\quad \,\,t > 0,$$

68 $$\theta_{1} \left( {1,t} \right) = \theta_{2} \left( {1,t} \right) = 1,\quad \,\,t > 0,$$

69 $$\left. {\frac{{\partial \theta_{1} \left( {y,t} \right)}}{\partial y}} \right|_{y = 1} = k_{0} \left. {\frac{{\partial \theta_{2} \left( {y,t} \right)}}{\partial y}} \right|_{y = 1} ,\,\,\quad t > 0.$$

Applying the Laplace transform to Eq. (65), we obtain the transformed equation

70 $$\frac{{\partial^{2} {{\overline{\theta}}}_{i} (y,s)}}{{\partial y^{2} }} - Q_{i} s{{\overline{\theta}}}_{i} (y,s) + {\overline{W}}_{i} (y,s) = 0,\quad \,\;i = 1,2,\,\,\,Q_{i} = \Pr_{i} {\text{Re}}_{i} .$$

The general solution of Eq. (70) is

71 $${{\overline{\theta}}}_{i} (y,s) = {\overline{D}}_{i1} (y,s)e^{{ - y\sqrt {Q_{i} s} }} + {\overline{D}}_{i2} (y,s)e^{{y\sqrt {Q_{i} s} }} ,$$

where ${\overline{D}}_{i1}$, ${\overline{D}}_{i2}$ have to satisfy conditions

72 $$\begin{gathered} e^{{ - y\sqrt {Q_{i} s} }} {\overline{D}}^{\prime}_{i1} (y,s) + e^{{y\sqrt {Q_{i} s} }} {\overline{D}}^{\prime}_{i2} (y,s) = 0, \hfill \\ - \sqrt {Q_{i} s} \,e^{{ - y\sqrt {Q_{i} s} }} {\overline{D}}^{\prime}_{i1} (y,s) + \sqrt {Q_{i} s} \,e^{{y\sqrt {Q_{i} s} }} {\overline{D}}^{\prime}_{i2} (y,s) = - {\overline{W}}_{i} (y,s). \hfill \\ \end{gathered}$$

From Eq. (72), we have

73 $${\overline{D}}^{\prime}_{i1} (y,s) = \frac{{e^{{y\sqrt {Q_{i} s} }} }}{{2\sqrt {Q_{i} s} }}{\overline{W}}_{i} (y,s);\,\,{\overline{D}}^{\prime}_{i2} (y,s) = \frac{{ - e^{{ - y\sqrt {Q_{i} s} }} }}{{2\sqrt {Q_{i} s} }}{\overline{W}}_{i} (y,s),$$

respectively,

74 $${\overline{D}}_{i1} (y,s) = \int\limits_{0}^{y} {\frac{{e^{{\xi \sqrt {Q_{i} s} }} }}{{2\sqrt {Q_{i} s} }}{\overline{W}}_{i} (\xi ,s} )d\xi + {\overline{E}}_{i1} (s),\,\,{\overline{D}}_{i2} (y,s) = - \int\limits_{0}^{y} {\frac{{e^{{ - \xi \sqrt {Q_{i} s} }} }}{{2\sqrt {Q_{i} s} }}{\overline{W}}_{i} (\xi ,s} )d\xi + {\overline{E}}_{i2} (s).$$

Replacing (74) into (71), we obtain

75 $${{\overline{\theta}}}_{i} (y,s) = {\overline{E}}_{i1} (s)e^{{ - y\sqrt {Q_{i} s} }} + {\overline{E}}_{i2} (s)e^{{y\sqrt {Q_{i} s} }} + e^{{ - y\sqrt {Q_{i} s} }} {\overline{\Phi }}_{i1} (y,s) + e^{{y\sqrt {Q_{i} s} }} {\overline{\Phi }}_{i2} (y,s),$$

where,

76 $${\overline{\Phi }}_{i1} (y,s) = \int\limits_{0}^{y} {\frac{{e^{{\xi \sqrt {Q_{i} s} }} }}{{2\sqrt {Q_{i} s} }}{\overline{W}}_{i} (\xi ,s} )d\xi ,\,\,{\overline{\Phi }}_{i2} (y,s) = - \int\limits_{0}^{y} {\frac{{e^{{ - \xi \sqrt {Q_{i} s} }} }}{{2\sqrt {Q_{i} s} }}{\overline{W}}_{i} (\xi ,s} )d\xi .$$

Unknown functions ${\overline{E}}_{i1} (s)$, ${\overline{E}}_{i2} (s)$ shall be determined using the boundary and interface conditions

77 $$\begin{gathered} {{\overline{\theta}}}_{1} (0,s) = 0,\,\,{{\overline{\theta}}}_{2} (d,s) = \frac{1}{s}, \hfill \\ {{\overline{\theta}}}_{1} (1,s) = \,\,{{\overline{\theta}}}_{2} (1,s), \hfill \\ \left. {\frac{{\partial {{\overline{\theta}}}_{1} \left( {y,s} \right)}}{\partial y}} \right|_{y = 1} = k_{0} \left. {\frac{{\partial {{\overline{\theta}}}_{2} \left( {y,s} \right)}}{\partial y}} \right|_{y = 1} . \hfill \\ \end{gathered}$$

After direct calculations, we obtain

78 $${\overline{E}}_{12} (s) = - {\overline{E}}_{11} (s),\,\,\,{\overline{E}}_{21} (s) = \frac{1}{s}e^{{d\sqrt {Q_{2} s} }} - {\overline{\Phi }}_{21} (d,s) - e^{{2d\sqrt {Q_{2} s} }} {\overline{\Phi }}_{22} (d,s) - e^{{2d\sqrt {Q_{2} s} }} {\overline{E}}_{22} (s),$$

79 $${\overline{E}}_{11} = \frac{ - 1}{2}\frac{{{\overline{G}}_{1} (s)\sqrt {Q_{2} s} \cosh \left[ {(d - 1)\sqrt {Q_{2} s} } \right] + {\overline{G}}_{2} (s)\sinh \left[ {(d - 1)\sqrt {Q_{2} s} } \right]}}{{\sqrt {Q_{2} s} \sinh \left( {\sqrt {Q_{1} s} } \right)\cosh \left[ {(d - 1)\sqrt {Q_{2} s} } \right] + \sqrt {Q_{1} s} \cosh \left( {\sqrt {Q_{1} s} } \right)\sinh \left[ {(d - 1)\sqrt {Q_{2} s} } \right]}},$$

80 $${\overline{E}}_{22} = \frac{1}{2}\frac{{{\overline{G}}_{1} (s)\sqrt {Q_{1} s} \cosh \left( {\sqrt {Q_{1} s} } \right) - {\overline{G}}_{2} (s)\sinh \left( {\sqrt {Q_{1} s} } \right)}}{{e^{{d\sqrt {Q_{2} s} }} \left\{ {\sqrt {Q_{2} s} \sinh \left( {\sqrt {Q_{1} s} } \right)\cosh \left[ {(d - 1)\sqrt {Q_{2} s} } \right] + \sqrt {Q_{1} s} \cosh \left( {\sqrt {Q_{1} s} } \right)\sinh \left[ {(d - 1)\sqrt {Q_{2} s} } \right]} \right\}}},$$

where,

81 $$\begin{aligned} {\overline{G}}_{1} (s) & = \frac{1}{s}e^{{(d - 1)\sqrt {Q_{2} s} }} - e^{{ - \sqrt {Q_{2} s} }} {\overline{\Phi }}_{21} (d,s) - e^{{(2d - 1)\sqrt {Q_{2} s} }} {\overline{\Phi }}_{22} (d,s) + e^{{ - \sqrt {Q_{2} s} }} {\overline{\Phi }}_{21} (1,s) + e^{{\sqrt {Q_{2} s} }} {\overline{\Phi }}_{22} (1,s), \\ {\overline{G}}_{2} (s) & = - \frac{{\sqrt {Q_{2} s} }}{s}e^{{(d - 1)\sqrt {Q_{2} s} }} + \sqrt {Q_{2} s} e^{{ - \sqrt {Q_{2} s} }} {\overline{\Phi }}_{21} (d,s) + \sqrt {Q_{2} s} e^{{(2d - 1)\sqrt {Q_{2} s} }} {\overline{\Phi }}_{22} (d,s) \\ & \quad - \sqrt {Q_{2} s} e^{{ - \sqrt {Q_{2} s} }} {\overline{\Phi }}_{21} (1,s) + \sqrt {Q_{2} s} e^{{\sqrt {Q_{2} s} }} {\overline{\Phi }}_{22} (1,s) + \sqrt {Q_{1} s} e^{{ - \sqrt {Q_{1} s} }} {\overline{\Phi }}_{11} (1,s) - \sqrt {Q_{1} s} e^{{\sqrt {Q_{1} s} }} {\overline{\Phi }}_{12} (1,s). \\ \end{aligned}$$

Numerical values of the temperature $\theta_{i} (y,t)$ could be determined with the numerical approximate formula (48) updated for ${{\overline{\theta}}}_{i} (y,s)$. However, the expressions of the temperature fields (75) are quite complicated and also contain functions $\Phi_{i1} (s),\;\Phi_{i2} (s)$ that are defined by means of some definite integrals. For this reason, the numerical inversion with the Stehfest's algorithm becomes difficult. Therefore, in the following, we will elaborate a numerical scheme adequate to the integration of the differential Eq. (65) with the initial-boundary conditions (66)–(69).

### Numerical scheme

Temperature fields $\theta_{1} (y,t),\;\theta_{2} (y,t)$ are solutions of the equations

82 $$\begin{gathered} Q_{1} \frac{{\partial \theta_{1} (y,t)}}{\partial t} - \frac{{\partial^{2} \theta_{1} (y,t)}}{{\partial t^{2} }} - W_{1} (y,t) = 0, \hfill \\ Q_{2} \frac{{\partial \theta_{2} (y,t)}}{\partial t} - \frac{{\partial^{2} \theta_{2} (y,t)}}{{\partial t^{2} }} - W_{2} (y,t) = 0, \hfill \\ \end{gathered}$$

along with the initial, boundary and interface conditions

83 $$\theta_{1} (y,0) = 0,\;y \in [0,1];\;\theta_{2} (y,0) = 0,\;y \in [1,d],$$

84 $$\theta_{1} (0,t) = 0;\;\theta_{2} (d,t) = 1,\;t > 0,$$

85 $$\begin{gathered} \theta_{1} (1,t) = \theta_{2} (1,t) = 0,\;t > 0, \hfill \\ \left. {\frac{{\partial \theta_{1} (y,t)}}{\partial y}} \right|_{y = 1} = \left. {\frac{{\partial \theta_{2} (y,t)}}{\partial y}} \right|_{y = 1} . \hfill \\ \end{gathered}$$

We consider the step size $\Delta t$ for the time t, step size $\Delta y_{1}$ for the spatial coordinate $y \in [0,1]$, and the sequences $t_{n} = n\Delta t,\;n = 0,1,...,N_{t} ,\;y_{j} = j\Delta y_{1} ,\;j = 0,1,...,N_{1} ,\;\Delta t = T/N_{t} ,\;\Delta y_{1} = 1/N_{1}$. Using the approximate formulas for the derivatives,

86 $$\begin{gathered} \left. {\frac{\partial \chi (y,t)}{{\partial t}}} \right|_{{(y,t) = (y_{j} ,t_{n} )}} = \frac{1}{\Delta t}\left[ {\chi (y_{j} ,t_{n + 1} ) - \chi (y_{j} ,t_{n} )} \right],n = 0,1, \ldots ,N_{t} - 1,j = 0,1, \ldots ,N_{1} , \hfill \\ \left. {\frac{{\partial^{2} \chi (y,t)}}{{\partial y^{2} }}} \right|_{{(y,t) = (y_{j} ,t_{n} )}} = \frac{1}{{\Delta y^{2} }}\left[ {\chi (y_{j + 1} ,t_{n} ) - 2\chi (y_{j} ,t_{n} ) + \chi (y_{j - 1} ,t_{n} )} \right],n = 0,1, \ldots ,N_{t} ,j = 1,2, \ldots N_{1} - 1, \hfill \\ \end{gathered}$$

Equation (82)_1_ is written as

87 $$\theta_{1}^{j,n + 1} = \alpha_{00} \theta_{1}^{j + 1,n} + \alpha_{01} \theta_{1}^{j,n} + \alpha_{02} \theta_{1}^{j - 1,n} + \alpha_{03} W_{1}^{j,n} ,\;n = 0,1, \ldots ,N_{t} - 1,\;j = 1,2, \ldots ,N_{1} - 1,$$

where,

88 $$\theta_{1}^{j,n} = \theta_{1} \left( {y_{j} ,t_{n} } \right),\;{\text{and}},\;\alpha_{00} = \alpha_{02} = \frac{\Delta t}{{Q_{1} \Delta y_{1}^{2} }},\;\alpha_{01} = 1 - \frac{2\Delta t}{{Q_{1} \Delta y_{1}^{2} }},\;\alpha_{03} = \frac{\Delta t}{{Q_{1} }}.$$

The initial and boundary conditions (83) and (84) lead to the following relations:

89 $$\theta_{1}^{j,0} = 0,\;j = 0,1,...,N_{1} ;\quad \;\theta_{1}^{0,n} = 0,\;n = 0,1,...,N_{t} .$$

For the interval $y \in [1,d]$, we consider the sequence

90 $$y_{{N_{1} }} = 1,\;y_{{N_{1} + k}} = 1 + k\Delta y_{2} ,\quad \;k = 1,2,...,N_{2} ,\;\Delta y_{2} = \frac{d - 1}{{N_{2} }}.$$

From the interface condition (85)_1_, we obtain

91 $$\theta_{1}^{{N_{1} ,n}} = \theta_{1}^{{N_{1} ,n}} ,\;\quad n = 0,1, \ldots ,N_{t} ,$$

while, from the condition (85)_2_, we have

92 $$\begin{gathered} \theta_{1}^{{N_{1} ,n}} = \theta_{2}^{{N_{1} ,n}} = \delta_{00} \theta_{2}^{{N_{1} + 1,n}} + \delta_{01} \theta_{1}^{{N_{1} - 1,n}} ,\quad \;n = 1,2, \ldots ,N_{t} , \hfill \\ \delta_{00} = \frac{{k_{0} \Delta y_{1} }}{{\Delta y_{2} + k_{0} \Delta y_{1} }},\delta_{01} = \frac{{\Delta y_{2} }}{{\Delta y_{2} + k_{0} \Delta y_{1} }}. \hfill \\ \end{gathered}$$

Equation (82)_2_ is written as

93 $$\theta_{2}^{{N_{1} + k,n + 1}} = \beta_{00} \theta_{2}^{{N_{1} + k + 1,n}} + \beta_{01} \theta_{2}^{{N_{1} + k,n}} + \beta_{02} \theta_{2}^{{N_{1} + k - 1,n}} + \beta_{03} W_{2}^{{N_{1} + k,n}} ,\quad \;n = 0,1,...,N_{t} - 1,\;k = 1,2,...,N_{2} - 1,$$

where,

94 $$\theta_{1}^{{N_{1} + k,n}} = \theta_{1} \left( {y_{{N_{1} + k}} ,t_{n} } \right),\;{\text{and}},\;\beta_{00} = \beta_{02} = \frac{\Delta t}{{Q_{2} \Delta y_{2}^{2} }},\;\beta_{01} = 1 - \frac{2\Delta t}{{Q_{2} \Delta y_{2}^{2} }},\;\beta_{03} = \frac{\Delta t}{{Q_{2} }}.$$

Using the boundary condition (84)_2_, we obtain

95 $$\theta_{2}^{{N_{1} + N_{2} ,n}} = 1,\quad \;n = 1,2,...,N_{t} .$$

Now, the above relations, determine the values $\theta_{1} (y_{j} ,t_{n} ),\;\theta_{2} (y_{k} ,t_{n} )$ of the temperature fields.

Indeed, using Eqs. (93) and (95), the values $\theta_{2}^{{N_{1} + k,n}} ,\;k = 1,2,...,N_{2} ,\;n = 0,1,...,N_{t}$ are determined.

The values $\theta_{1}^{j,n} ,\;j = 0,1,...,N_{1} ,\;n = 0,1,...,N_{t}$ of the temperature field in zone $y \in [0,1]$ are determined using Eq. (87) together with Eq. (92).

## Numerical results and discussion

Flows inside channels, such as magneto-hydrodynamic, electro-hydrodynamic, and electrokinetic flows are important in many microscale applications. The generation of such flows is often done with the help of external fields (mechanical, thermal, magnetic, electric) applied to the fluids. In this section, the generalized Couette flow studied in previous paragraphs is numerically and graphically simulated in the case when the motion of the upper plate is given by the function $f(t) = 1 - \exp ( - t),\;t \ge 0$.

For the non-dimensional parameters that characterize the studied fluids and fields, the following numerical values were used: $$d = 1.5,\;\omega = \pi /4,\;F_{1} = 0.5,\;F_{2} = 0.6,$$$$E_{1} = 0.55,$$ $$E_{2} = 0.75,\;Re_{1} = 0.8,\;\;Re_{2} = 2,\;Rm_{1} = 1.2,\;Rm_{2} = 0.8,\;Pr_{1} = 1.5,\;Pr_{2} = 3.5,$$$$Ec_{1} = 2.5,\;Ec_{2} = 1.5,$$ $$G_{0} = 1.5,\;Hb_{1} = 0.8,\;Hb_{2} = 0.4,\;\gamma_{0} = \pi /6$$.

**Figure 2:**
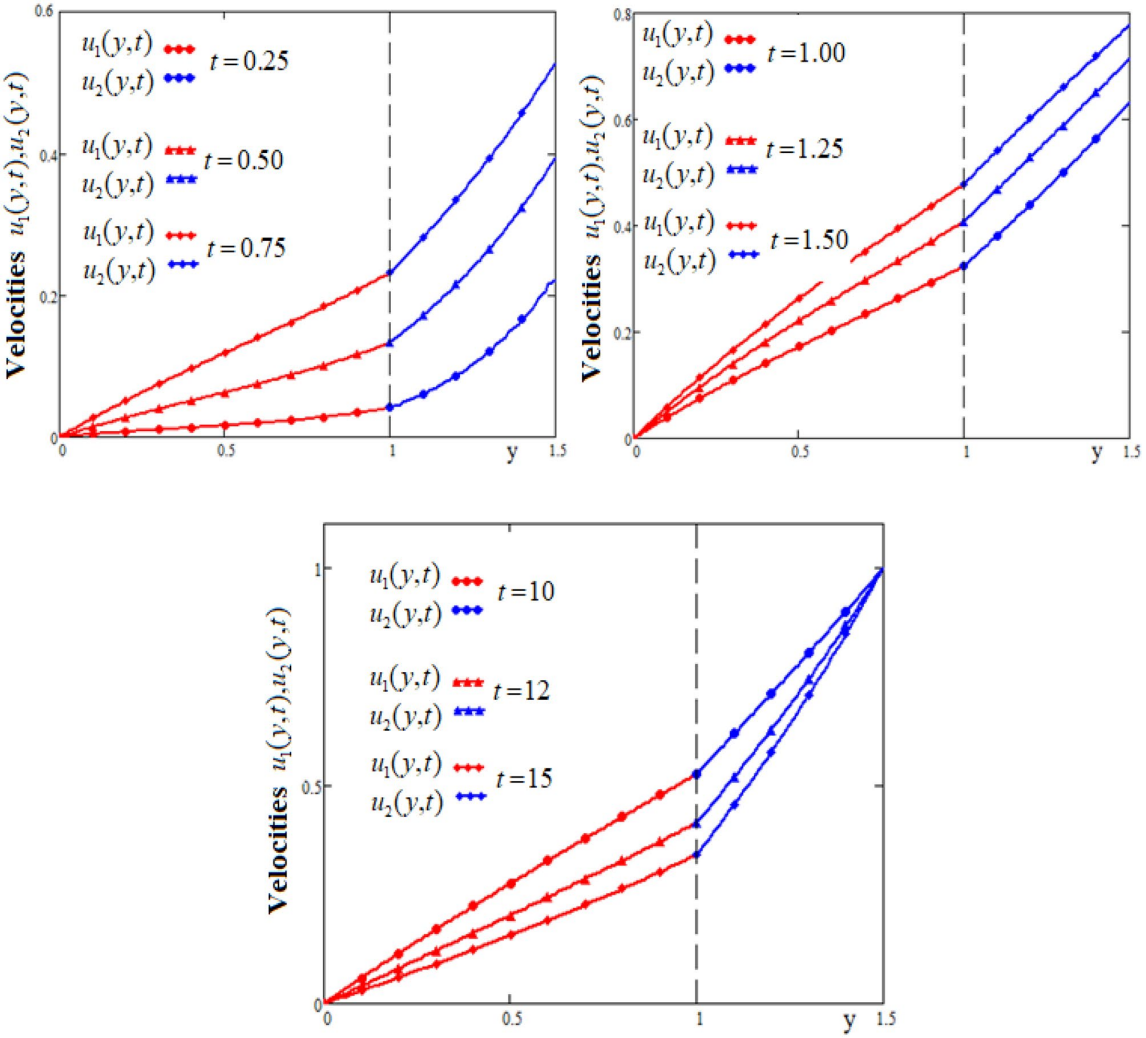
The profiles of velocities $u_{1} (y,t),\;u_{2} (y,t)$ versus y for different values of the time t.

Figure 2 shows the profiles of fluid velocities in both flow regions for different values of the time t. As expected, the fluid flows more slowly in the region bounded by the lower wall. This behavior is due to the fact that the lower wall of the canal is fixed, so the viscosity force will slow down the fluid motion. In the region of the canal bounded by the upper wall, the fluid moves at a higher velocity due to the movement of the wall with the velocity given by the function $f(t)$. It is observed in Fig. 2 that for large values of the time t, fluid velocity on the upper wall becomes almost constant, because $\lim_{t \to \infty } f(t) = \lim_{t \to \infty } (1 - \exp ( - t)) = 1$.

**Figure 3:**
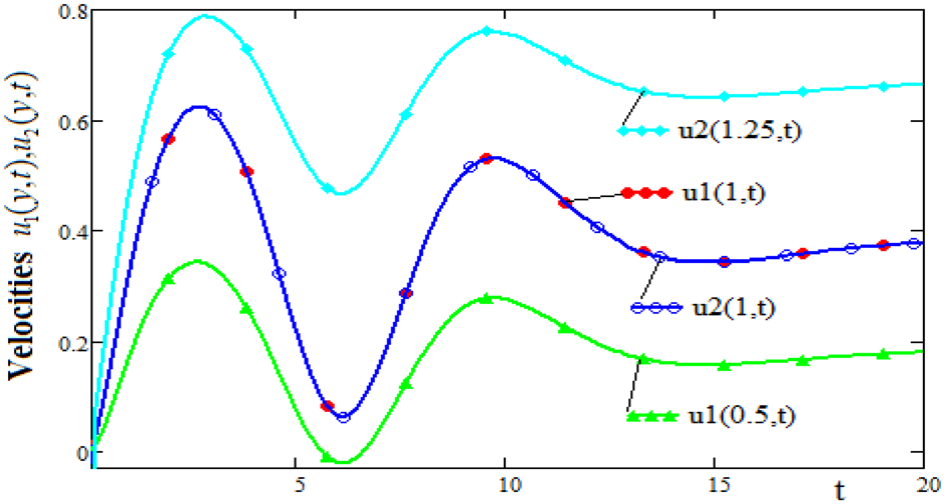
Time-evolution of velocities $u_{1} (y,t),\;u_{2} (y,t)$ in three different positions of channel.

This property is most clearly evident from Fig. 3 which shows the profiles of velocities as a function of time t for three positions of the channel, namely, $y \in \{ 0.5,1.0,1.25\}$.The curves in Fig. 2, show that for $t \ge 15$ fluid velocities tend to become constant. Also, Figs. 2 and 3 highlight that fluid velocity satisfy the interface condition $u_{1} (1,t) = u_{2} (1,t)$.

**Figure 4:**
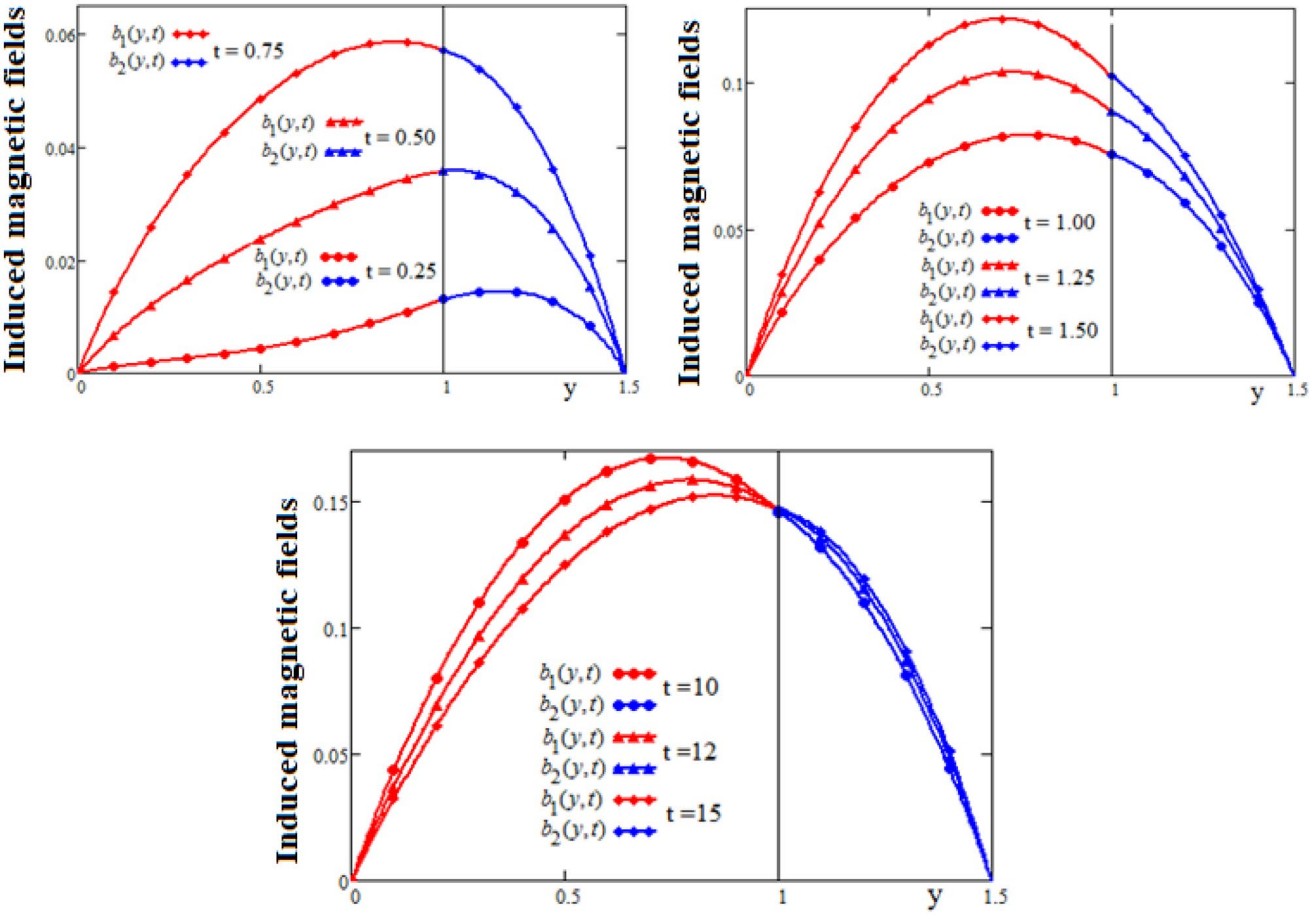
The profiles of induced magnetic fields $b_{1} (y,t),\;b_{2} (y,t)$ versus y for different values of the time t.

**Figure 5:**
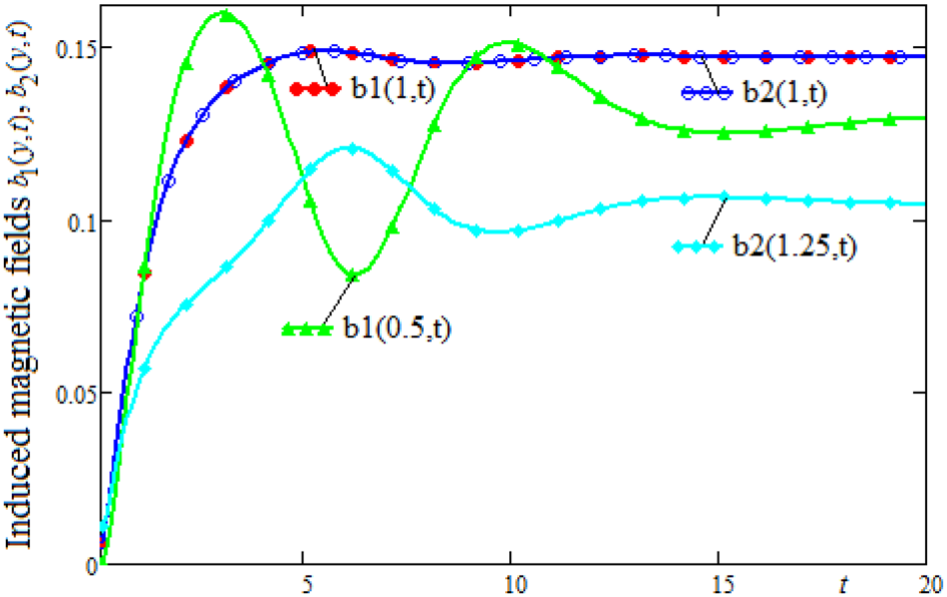
Time-evolution of induced magnetic fields $b_{1} (y,t),\;b_{2} (y,t)$ in three different positions of channel.

Figures 4 and 5 show profiles of the ratio of induced magnetic field, and the external applied magnetic field, versus spatial coordinate y, respectively versus time t. Note that in the $y = 1$ position of the channel, where the velocities are equal, the variation of the induced magnetic field is slower. It can be seen that in this position the induced magnetic field has a slow increase and after the value $t = 5$, the values of the induced magnetic field are almost constant. In the first flow zone, in which the velocity gradient has large variations, the induced magnetic field also has significant variations. These variations are attenuated in the second flow area where the speed gradient variations are smaller. Also, we note that for $t \ge 15$, the values of induced magnetic field become almost constant for any position of the channel. This behavior is in accordance with the fluid motion whose velocity tends to constant values for large values of the time t.

**Figure 6:**
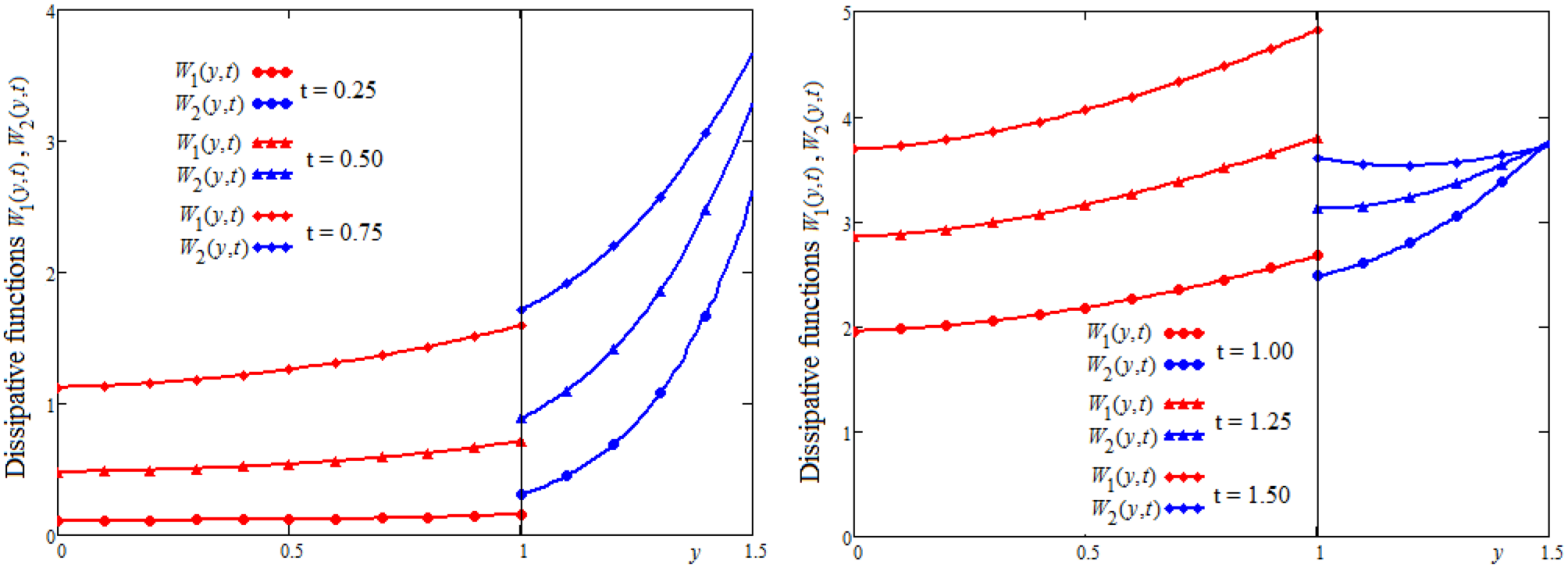
The profiles of dissipative functions $W_{1} (y,t),\;W_{2} (y,t)$ versus y for different values of the time t.

**Figure 7:**
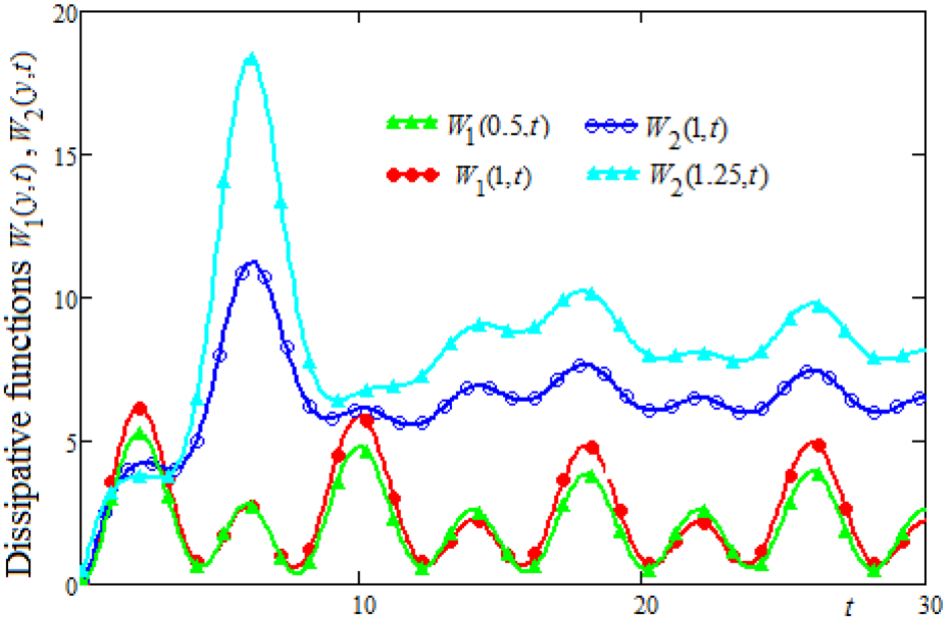
Time-evolution of the dissipative functions $W_{1} (y,t),\;W_{2} (y,t)$ in three positions of channel.

Figures 6 and 7 are plotted to show the variation in space and time of the dissipative functions $W_{1} (y,t),\;W_{2} (y,t)$. It is known that viscous dissipation acts as a source of energy in the fluid flow and it affects the temperature distribution. The shear stress within the fluid layer induced by the motion of the upper plate significantly influences the energy dissipation. It is observed in Figs. 6 and 7 that for a short time interval, the dissipative function in the region bounded by the lower wall is higher than the dissipative function in the other region. After this instant, the dissipative function in the region bounded by the upper plate becomes higher. This fact is generating by the evolution in time of the velocity in this region.

## Conclusions

Unsteady, magneto-hydrodynamic generalized Couette flows of two immiscible fluids in a rectangular channel have been studied.

The flow is influenced by an inclined magnetic field and an axial time-oscillating electric field. Both fluids are considered electrically conducting and the solid boundaries are electrically insulated.

The problem is formulated into the dimensionless form and at the interface the velocity, shear stress, induced magnetic and temperature fields are considered continuous functions. Both walls are isothermal. The bottom wall of channel is fixed, while the upper wall is moving with a given time-dependent velocity. The nonslip conditions on the solid boundaries are also considered.

Approximate analytical solutions for the velocity, induced magnetic, and temperature fields have been determined using the Laplace transform method along with the numerical Stehfest's algorithm for the inversion of the Laplace transforms.

A numerical scheme based on the finite differences has been developed for the non-linear energy equation.

A particular case characterized by a time-exponential velocity of the upper wall, has been numerically and graphically studied to show the evolution of the fluid velocity, induced magnetic field, and viscous dissipation in both flow regions.
